# CircHIPK3 promotes proliferation and metastasis and inhibits apoptosis of renal cancer cells by inhibiting MiR-485-3p

**DOI:** 10.1186/s12935-020-01319-3

**Published:** 2020-06-16

**Authors:** Jinjin Lai, Jun Xin, Changde Fu, Wei Zhang

**Affiliations:** Department of Urology, Quanzhou First Hospital Affiliated to Fujian Medical University, 248-252 East Street, Licheng District, Quanzhou, 362002 Fujian Province China

**Keywords:** Renal carcinoma, circHIPK3, miR-485-3p, Epithelial-mesenchymal transition, Apoptosis

## Abstract

**Background:**

The intervention of circHIPK3 in renal carcinoma (RC) has not been reported, and thus, the current study investigated the intervention and mechanism of circHIPK3 in RC.

**Methods:**

The expression of circHIPK3 in RC tissues and cells was detected by quantitative real-time polymerase chain reaction (qRT-PCR). Ribonuclease R (RNase R) resistance and distribution of circHIPK3 and HIPK3 were analyzed by RNase R digestion experiments and cytoplasm/nucleus separation experiments. CircHIPK3 was knocked down in ACHN and 769-P cells. Cell counting kit-8 (CCK-8), colony formation assay, scratch assay, and Transwell assay were performed to detect cell proliferation and metastasis. CircInteractome, qRT-PCR and dual-luciferase reporter assay were used to predict the target miRNAs of circHIPK3. Furthermore, a series of rescue experiments were performed to analyze the regulatory relationship between circHIPK3 and miR-485-3p. Epithelial-mesenchymal transition (EMT) and the expressions of apoptosis-associated markers were detected by Western blot and qRT-PCR. The regulatory relationship between circHIPK3 and miR-485-3p in vivo was explored by xenograft experiments, Western blot, qRT-PCR and immunohistochemistry (Ki-67).

**Results:**

CircHIPK3 was mainly overexpressed in the cytoplasm of RC tissues and cells. Knocking down circHIPK3 inhibited the proliferation, migration, and invasion of RC cells. The expression of circHIPK3 was negatively related to that of its target gene miR-485-3p. Results of the rescue experiments showed that circHIPK3 overexpression could partially reverse the anti-carcinoma effect of miR-485-3p mimic. The specific mechanism of circHIPK3 was related to the effect of miR-485-3p on partially reversing the up-regulated expressions of Clever caspase-3, Bax, E-Cadherin and down-regulated expressions of Bcl-2, N-Cadherin and Vimentin. The results of in vivo experiments demonstrated that circHIPK3 promoted tumor growth and the expression of Ki-67 by down-regulating miR-485-3p.

**Conclusion:**

CircHIPK3 promotes the proliferation and metastasis and inhibits the apoptosis of RC cells through competitively binding to miR-485-3p.

## Background

Renal carcinoma (RC) originates from renal tubular epithelial cells in the renal parenchyma, and it is one of the most common malignant tumors of the urinary system. In recent years, the incidence of RC showed an increasing trend worldwide [1]. Statistics demonstrated that about 20% of RC patients had tumor metastasis at first diagnose, and recurrent metastasis occurred in nearly 30% of patients with localized RC after surgical resection [2, 3]. At present, main clinical treatments for RC are surgical resection in combination with immunotherapy, and targeted drug therapy. Molecular-targeted therapy, as a current research hotspot in refractory diseases such as carcinoma, can specifically reduce lesions and drug damage to normal tissues with the advantages of high efficiency and low toxicity for disease prevention and treatment [4, 5]. Although the current molecular-targeted therapy has effectively improved the progression and prognosis of RC, the continuous use of drugs with single targets in the meantime has greatly enhanced drug resistance [6]. Therefore, researchers are currently devoted to discovering molecular-targeted drugs acting on different targets in order to reduce drug resistance and improve efficacy.

Non-coding RNAs (ncRNAs) account for 98.5% of all non-protein coding sequences of human genomes [7]. NcRNAs, a breakthrough in molecular-targeted therapy, are involved in the regulation of almost all physiological or pathological processes such as embryonic development, cell differentiation, apoptosis, metabolism, signaling, infection, and immune response [8]. Circular RNAs (circRNAs) belong to ncRNAs and are formed in reverse splicing during RNA maturation [9]. The upstream 5′ end binding site and the downstream 3′ end binding site are reversely connected to form a circle, which replaces the traditional linear connection [10]. The wide distribution and high abundance of circRNA in cells were gradually discovered in the early 21st century, and its advantages are recognized to include the prevention and treatment of various diseases. For example, circANRIL induces nucleolar stress and activates p53, thus resulting in atherosclerosis [11]; circS-7 (CDR1as), which has high expression in human brain and retina, is considered a biomarker for the diagnosis of Alzheimer’s disease and a therapeutic target for the treatment of the disease [12]; in addition, circRNA plays a key role in the occurrence and development of various carcinomas such as gastric carcinoma, bladder carcinoma, and liver carcinoma [13, 14]. However, the expression profile and function of circRNAs in RC remain unclear.

CircHIPK3, the second exon of Homeodomain-interacting protein kinase 3 (HIPK3), is high-expressed in the liver, brain, and lung [15]. HIPK3 is a serine/threonine protein kinase in cells that negatively regulates cell apoptosis through phosphorylation of molecules such as JUN and RUNX2 [16, 17]. Studies have shown that circHIPK3 interferes with the proliferation and metastasis of carcinomas such as colorectal caricinoma, bladder carcinoma, and prostate carcinoma, and it is a prognostic marker for gliomas [18–21]. However, little is known about the role of circHIPK3 in RC, and thus, the current study aimed to explore the intervention of circHIPK3 with RC and the underlying mechanisms, hoping to provide a strong basis for molecular-targeted therapy of RC.

## Methods

### Ethics statement and specimen collection

All clinical tissue specimens used in this experiment were obtained from Department of Urology of Quanzhou First Hospital Affiliated to Fujian Medical University. All the patients or their families signed informed consent. All studies involving human specimens were approved by the Ethics Committee of the Quanzhou First Hospital Affiliated to Fujian Medical University prior to the initiation of the study (approval number: 201601007MNWK). RC tissues (n = 48) and adjacent tissues (n = 48) were collected from RC patients who had received radical resection of RC in the Department of Urology of Quanzhou First Hospital Affiliated to Fujian Medical University from Jan 2016 to Dec 2018. The RC tissues and adjacent tissues were washed with pre-chilled normal saline, and then frozen in liquid nitrogen. Animal experiments in this study were conducted in strict accordance with relevant management regulations and operating procedures of animal experiments, and the experiments were approved by the Quanzhou First Hospital Affiliated to Fujian Medical University Ethics Committee (approval number: 201811023MNWK).

### Cell culture

It was reported that clear renal cell carcinoma accounts for almost 80% of all renal carcinomas, however, all types of renal carcinoma cells were commonly studied [5, 22, 23]. Human renal tubular epithelial cell HK-2 (ATCC^®^ CRL-2190™), clear renal cell carcinoma cell Caki-1 (ATCC^®^ HTB-46™), papillary renal carcinoma cell ACHN (ATCC^®^ CRL-1611™), Renal Carcinoma Cell 786-O (ATCC^®^ CRL-1932™), Renal Carcinoma Cell 769-P (ATCC^®^ CRL-1933™), and Renal Carcinoma Cell A498 (ATCC^®^ HTB-44™) were purchased from American Type Culture Collection (ATCC). The cells were maintained in culture flasks containing 10% Fetal Bovine Serum (FBS, ATCC^®^ 30-2021™) and corresponding medium according to the instructions, and then incubated in an incubator with 5% CO_2_ at 37 °C. The medium was changed every 2 days. HK-2 cells were cultured in Defined Keratinocyte SFM (K-SFM, 10744019, Gibco, USA), while Caki-1 cells were cultured in McCoy’s 5A Medium (ATCC^®^ 30-2007™). Both ACHN and A498 cells were cultured in Eagle’s Minimum Essential Medium (EMEM, ATCC^®^ 30-2003™), and 786-O cells were cultured in RPMI-1640 medium (21875-091, GIBCO, USA).

### Nuclear/cytoplasmic separation and ribonuclease R (RNase R) digestion experiments

To analyze the resistance of circHIPK3 to RNase R and the abundance of circHIPK3 in the cytoplasm and nuclei, we performed cytoplasm/nucleus separation and RNase R digestion experiments. ACHN and 769-P RC cells were seeded in 6-well plates (2 negative wells) at 2 × 10^5^ cells/well. The cells were digested using 0.25% Trypsin–EDTA Solution (ATCC^®^ 30-2101™, USA) when they reached 80% confluence. Then the cells were centrifuged at 2000×*g* for 2 min at 4 °C. Next, cytoplasmic and nuclear RNAs were extracted using Minute (TM) Cytoplasmic and Nuclear Fractionation kit according to the instructions (SC-003, INVENT, USA). The extracted RNAs were divided into two groups, namely, RNase digestion group (RNase+) and RNase non-digestion group (RNase−). The reaction system consisted of 4 μg of RNA, 4 U of RNase R (R4875, Sigma-aldrich, Germany), 2 μl of 10 × Reaction Buffer, and 20 μl of RNase-Free Water. The above mixture was reacted at 37 °C for 10 min. Subsequently, RNAs of the digested products were extracted by phenol/chloroform and ethanol precipitation, and reverse-transcribed into cDNAs. The expressions of circHIPK3 and HIPK3 were detected by quantitative real-time polymerase chain reaction (qRT-PCR).

### Total RNA extraction and qRT-PCR

Total RNA extraction reagent Trizol (15596-026, Invitrogen, USA) was used to extract total RNAs from RC tissues or cells. RC tissues and adjacent carcinoma tissues were cut into 100 mg slices and added with 1 ml of Trizol. After the tissues were thoroughly ground and centrifuged, the supernatant was separated. Next, chloroform (40064961, HUSHI, China) and isopropanol (I811925-500 ml, Macklin, China) were added to the supernatant to extract total RNAs. Total RNAs of the cells were extracted in a similar way only without the grinding process, and then reverse-transcribed into cDNAs using #k1622 RevertAidFirst Strand cDNA Synthesis Kit (Thermo Scientific). The cDNAs (tenfold) and primers were diluted with DEPC water. The qRT-PCR reaction system consisted of 2 μl of diluted cDNA, 6 μl of DEPC water, 2 μl of diluted primers, and 10 μl of SYBR reagent (04913914001, Roche, Swit) and the reaction was performed using Veriti™ 96-Well Fast Thermal Cycler (ThermoFisher, USA) under the following conditions: pre-denaturation at 95 °C for 10 min, denaturation at 95 °C for 15 s, annealing at 60 °C for 1 min, for 40 cycles. The relative expression levels of the mRNAs were determined by 2^−ΔΔCT^ method [24]. The primer sequences were as follows: circHIPK3-F, 5′-GGATCGGCCAGTCATGTATC-3′, and circHIPK3-R, 5′-ACCGCTTGGCTCTACTTTGA-3′; HIPK3-F, 5′-CATATCTACAATCTCGGTACTACAGAGC-3′, and HIPK3-R, 5′-GTATCGAATCTGATCATACTCCAAGGCTC-3′; miR-338-3p-F, 5′-TGTTGGTCGTATCCAGTGCAA-3′, and miR-338-3p-R, 5′-GTATCCAGTGCGTGTCGTGG-3′; miR-375-F, 5′-AACCGTCGTATCCAGTGCAA-3′, and miR-375-R, 5′-GTCGTATCCAGTGCGTGTCG-3′; miR-485-3p-F, 5′-TACACGGCTCTCCTCTCTGT-3′, and miR-485-3p-R, 5′-TGTCGTGGAGTCGGCAATTG-3′; miR-495-F, 5′-TTCGGTCGTATCCAGTGCAA-3′, and miR-495-R, 5′-GTCGTATCCAGTGCGTGTCG-3′; Bcl-2-F, 5′-GAAGCACAGATGGTTGATGG-3′, and Bcl-2-R, 5′-CAGCCTCACAAGGTTCCAAT-3′; Bax-F, 5′-CACAACTCAGCGCAAACATT-3′, and Bax-R, 5′-ACAGCCATCTCTCTCCATGC-3′; CDH1 for E-Cadherin (E-Cad)-F, 5′-GTGAACACCTACAATGCCGC-3′, and E-Cad-R, 5′-CCCAGGGGACAAGGGTATGA-3′; CDH2 for N-Cadherin (N-Cad)-F, 5′-AGGCTTCTGGTGAAATCGCA-3′, and N-Cad-R, 5′-GGAGGGATGACCCAGTCTCT-3′; VIM for Vimentin-F, 5′-TCTGGTCTAACGGTTTCCCCT-3′, and Vimentin-R, 5′-TTCAAGGTCAAGACGTGCCA-3′; U6-F, 5′-GCGCGTCGTGAAGCGTTC-3′, and U6-R, 5′-GTGCAGGGTCCGAGGT-3′; glyceraldehyde-3-phosphate dehydrogenase (GAPDH)-F, 5′-GGGTGTGAACCATGAGAAGTATGAC-3′, and GAPDH-R, 5′-GTGGTCATGAGTCCTTCCACGATACC-3′. GAPDH and U6 were used as internal references in this experiment.

### Cell Counting Kit-8 (CCK-8) assay

CCK-8 assay was performed to detect the changes of the viability of RC cells. The concentrations of ACHN and 769-P RC cells after digestion were adjusted to 1 × 10^3^ cells/ml, The cells (100 μl) were inoculated to 96 wells in duplicate plates, and then incubated in an incubator with 5% CO_2_ at 37 °C for 48 h. Afterwards, 10 μl/well of CCK-8 reagent (CA1210, Solarbio, China) was added to the cells in each well. Next, the absorbance of the cells was measured at 450 nm using an iMark microplate reader (BIO-RAD, USA) after incubation for 24, 48, and 72 h.

### Colony formation assay

The proliferation of ACHN and 769-P RC cells was measured by colony formation assay. Then, the cells were digested with 0.25% Trypsin–EDTA Solution to prepare a cell suspension. Next, the cells of each group were seeded into 6-well plates at a concentration of 5 × 10^2^ cells/well, and then incubated at 37 °C in 5% CO_2_ for 2 weeks. Afterwards, the cells were washed with PBS, fixed with methanol for 15 min, and stained with Giemsa staining solution (32884, Sigma, USA) for 20 min. Next, the stained cells were washed with distilled water and dried at room temperature. Finally, the cells were counted under a microscope to determine the number of cell colonies.

### Scratch assay

The back of the 6-well plate was scratched with horizontal lines spaced 1 cm apart. The ACHN and 769-P RC cells to be tested after digestion were prepared in a cell suspension, and 5 × 10^5^ cells were added to each well of the plate. After incubation for 24 h, we used a pipette to make perpendicular scratch on the horizontal lines and removed the cells inside. Next, a corresponding medium without serum was added to the cells and cultured at 37 °C in 5% CO_2_. Cell migration was observed under a microscope 100 times at 0 and 24 h.

### Transwell assay

The effects of circHIPK3 and miR-485-3p on the invasion of ACHN and 769-P RC cells were observed by Transwell assay. A medium containing 0.1% FBS was added to the digested cells. Then the pre-treated and digested cells (1 × 10^4^ cells) of each group were seeded into the upper chamber of the Transwell (8 μm, BD Biosciences, USA) that had been pre-coated with Matrigel gel, followed by culture for 24 h. Next, the transwell chamber was removed and the medium was discarded. Thirty min after being fixed with 4% paraformaldehyde, the invaded cells were stained with 0.1% crystal violet. Finally, the numbers of the invaded cells of different treatment groups were counted under a microscope 250 times.

### CircInteractome

The circInteractome database (https://circinteractome.nia.nih.gov/index.html) was used to predict the binding sites for RNA on circRNA in circbase, while Targetscan was used to predict the potential binding sites for miRNAs and circRNA. CircInteractome database revealed that circHIPK3 can target miR-338-3p, miR-375, miR-485-3p, and miR-495.

### Dual-luciferase reporter assay

Dual-luciferase reporter assay was conducted to verify the targeting relationship between circHIPK3 and miR-485-3p. The pmirGLO plasmid (CL414-01, Biomed, China) was constructed. The cells were divided into following eight groups: circHIPK3-wild-type (WT) + miR-485-3p mimic control group, circHIPK3-WT + miR-485-3p mimic group, circHIPK3-mutant (mut) + miR-485-3p mimic control group, circHIPK3-mut + miR-485-3p mimic group, circHIPK3-WT + miR-485-3p inhibitor control group, circHIPK3-WT + miR-485-3p inhibitor group, circHIPK3-mut + miR-485-3p inhibitor control group, and circHIPK3-mut + miR-485-3p inhibitor group. The cells were washed with PBS 24 h after transfection. Then 150 μl of 1 × Lysis Buffer PLB Reagent (Invitrogen, 1168-019, USA) was added into each well of the 24-well plate and incubated with the cells at room temperature for 20 min to fully lyse the cells. Fifty μl of Luciferase Assay Reagent II (LAR II) was then added to 10 μl of the cell lysate. Promega GLOMAX 20/20 (USA) was used to detect firefly luciferase activity. Next, 50 μl of Stop & Glo reagent was added to the mixture, and the Renilla luciferase activity of the cells were detected using Promega GLOMAX. Renilla luciferase served as an internal reference, and the activation degrees of different reporter genes were compared according to the ratio of firefly/Renilla.

### Plasmid construction and cell transfection

The circular RNA overexpression vector pLCDH-circRNA was purchased from Guangzhou GENESEED Biological Co., Ltd. (Cat. No. GS0104). The sequence of circHIPK3 (CirBase ID: hsa_circ_0000284) was obtained from the CircBase database (http://www.circbase.org/). The circHIPK3 sequence was amplified and the overexpression plasmid pLCDH-circHIPK3 was constructed. Next, competent cells DH5α were infected with pLCDH-circHIPK3 by lentivirus transfection method, and 293T cells were infected by lentivirus packaging. After 48 h, the cell lines were screened with 1 μg/ml puromycin. The supernatants of the virus containing pLCDH-circHIPK3 were collected and incubated with ACHN and 769-P cells to obtain stably transfected cell lines. Silent circHIPK3 (sicircHIPK3), miR-485-3p mimic (miR10002176-1-5), and miR-485-3p inhibitor (miR20002176-1-5) were purchased from China RIBOBIO Company. SicircHIPK3 target sequence was 5′-CUACAGGUAUGGCCUCACA-3′, and the miR-485-3p mature sequence was 5′-GUCAUACACGGCUCUCCUCUCU-3′. In addition to the overexpression plasmid, sicircHIPK3, miR-485-3p mimic (or mimic control), and miR-485-3p inhibitor (or inhibitor control) were transferred into the RC cells using Lipofectamine™ 3000 kit (L3000008, ThermoFisher, USA).

### Flow cytometry

Cell apoptosis was detected by flow cytometry. Cell suspension was prepared, and the cells were fixed with 70% ethanol at 4 °C overnight. The cells were then added with 5 ul of Annexin V-FITC reagent (C1062M, Beyotime, China) and 10 μl of propidium iodide reagent (PI, C1062M, Beyotime, China), and the mixture was thoroughly mixed and kept in the dark at room temperature for 20 min. Finally, the mixture was subjected to detection by Backman Coulter CytoFLEX flow cytometer.

### Western blot

Western blot was performed to detect the protein expressions of genes. The proteins were extracted from the cells. The cells were added with 100 μl of cell lysate (C874793, Macklin, China), and the lysate was fully lysed using a pipette. The solution was centrifuged for 10 min (1600×*g*, 4 °C), and the protein concentration was determined by BCA method. Then, 100 μg of the proteins were separated by sodium dodecyl sulfate–polyacrylamide gel electrophoresis (SDS-PAGE) and transferred to methanol-soaked PVDF membranes (IPVH00010, Millipore, USA). After being washed 3 times with 1 × TBST (10 min/time), the PVDF membranes were cut into strips and incubated with a blocking solution containing 5% skimmed milk powder for 1.5 h. After the PVDF membranes were washed again with 1 × TBST, different primary antibodies (C Caspase-3, ab2302, 1:1000; Bcl-2, ab59348, 1:800; Bax, ab32503, 1:1000; E-cadherin, ab40772, 1:10000; N-cadherin, ab18203, 1:1000; Vimentin, ab92547, 1:1000; GAPDH, ab8245, 1:10000) were added to the membranes and incubated overnight (4 °C). The membranes were washed again using 1 × TBST, and then the secondary antibody (Anti-Mouse, ab6728, 1:10000; Anti-Rabbit, ab6721, 1:10000) was added to further incubate with the membranes. After incubation for 1 h, the secondary antibodies were washed off. The PVDF membranes with ECL luminescent liquid (WBKLS0100, Millipore, Germany) were placed in a Bio-rad GelDoc XR gel imager. Image J was used to to analyze the gray values. GAPDH served as an internal reference in this study. All antibodies were purchased from Abcam (UK).

### Nude mouse tumor formation experiment

Twenty-four female BALB/C nude mice (SPF grade, weighing 18-20 g and aged 5 weeks old) were purchased from Beijing Charles River Animal Co., Ltd. (China). The nude mice were bred and the animal experiments were conducted in Quanzhou First Hospital Affiliated to Fujian Medical University SPF-level experimental animal centers, and the mice were provided with free access to food and water. The tumor formation experiment was initiated 3 d after the adaptive breeding. Twenty-four experimental nude mice were divided into 8 groups, namely, ACHN cells + negative control (NC) + miR-485-3p mimic control (MC) group, ACHN cells + CircHIPK3 (circ) + MC group, ACHN cells + NC + miR-485-3p mimic (M) group, ACHN cells + circ + M group; 769-P cells + NC + MC group, 769-P cells + circ + MC group, 769-P cells + NC + M group, and 769-P cells + circ + M group, with 3 mice in each group. Disposable syringe (1 ml) was subcutaneously injected via the right armpit into each nude mouse. Stably transfected ACHN cells and 769-P cells containing circHIPK3 overexpression plasmid, miR-485-3p agomir (miR40003129-4-5, Ruibo Biotechnology, China) and miR-485-3p agomir NC (miR4N0000001-4-5, Ruibo Biosciences, China) were injected into the nude mice of the corresponding groups (injection dose: 0.2 ml). The injection sites were disinfected with ethanol before injection, and the needles were slowly pulled out of the mice to prevent the injection fluid from overflowing. The injection was performed every 7 days (0.2 ml/d) to ensure the stability of the injection.

### Tumor weight

Two weeks after injection, tumors of all the nude mice were removed after anesthetizing the mice (50 mg/kg) using 0.5% sodium pentobarbital solution (P3761-25G, Sigma, USA) with physiological saline. After the muscle reaction of the extremities of the mice disappeared, the mice were fixed on the operating table in the supine position. Then, the right underarm skin was cut off with a sterilized surgical instrument to be fully exposed, and the transplanted tumor was quickly removed. The collected xenografts were weighed and photographed.

### Immunohistochemistry

The transplanted tumors were fixed with 4% paraformaldehyde, subjected to gradient ethanol dehydration, embedded in paraffin, and sectioned into 5-μm thick slices. After being deparaffinized and hydrated with xylene and ethanol respectively, the sections were immersed in 0.01% Triton X-100 solution (10789704001, Roche, Switzerland) for 10 min and in 3% H_2_O_2_ solution for 10 min, and then blocked with 10% goat serum for 1 h at 37 °C. Next, Anti-Ki67 antibody (ab15580, 1:500, Abcam, UK) was added and incubated with the sections overnight at 4 °C. The next day, the sections were washed with PBS and co-incubated with the corresponding secondary antibodies (ab6721, 1:10000, Abcam, UK) in a wet box for 1.5 h, and then stained with DAB and hematoxylin. After dyeing, the sections were rinsed in distilled water to remove the dye solution. Finally, the sections were subjected to gradient ethanol dehydration, transparentized with xylene, and sealed with neutral gum. The sections were observed under a microscope 500 times.

### Statistical analysis

Differences between two groups were compared using Student’s two-tailed *t* test, while differences between multiple groups were compared using one-way ANOVA or two-way ANOVA followed by Turkey’s t-test. Correlation analysis between miR-485-3p and circHIPK3 was performed using Pearson correlation coefficient. ROC curves were shown in survival analysis. *P *< 0.05 was considered as statistically significant.

## Results

### CircHIPK3 was up-regulated in RC tissues and cells, and was not sensitive to RNase R

**Fig. 1:**
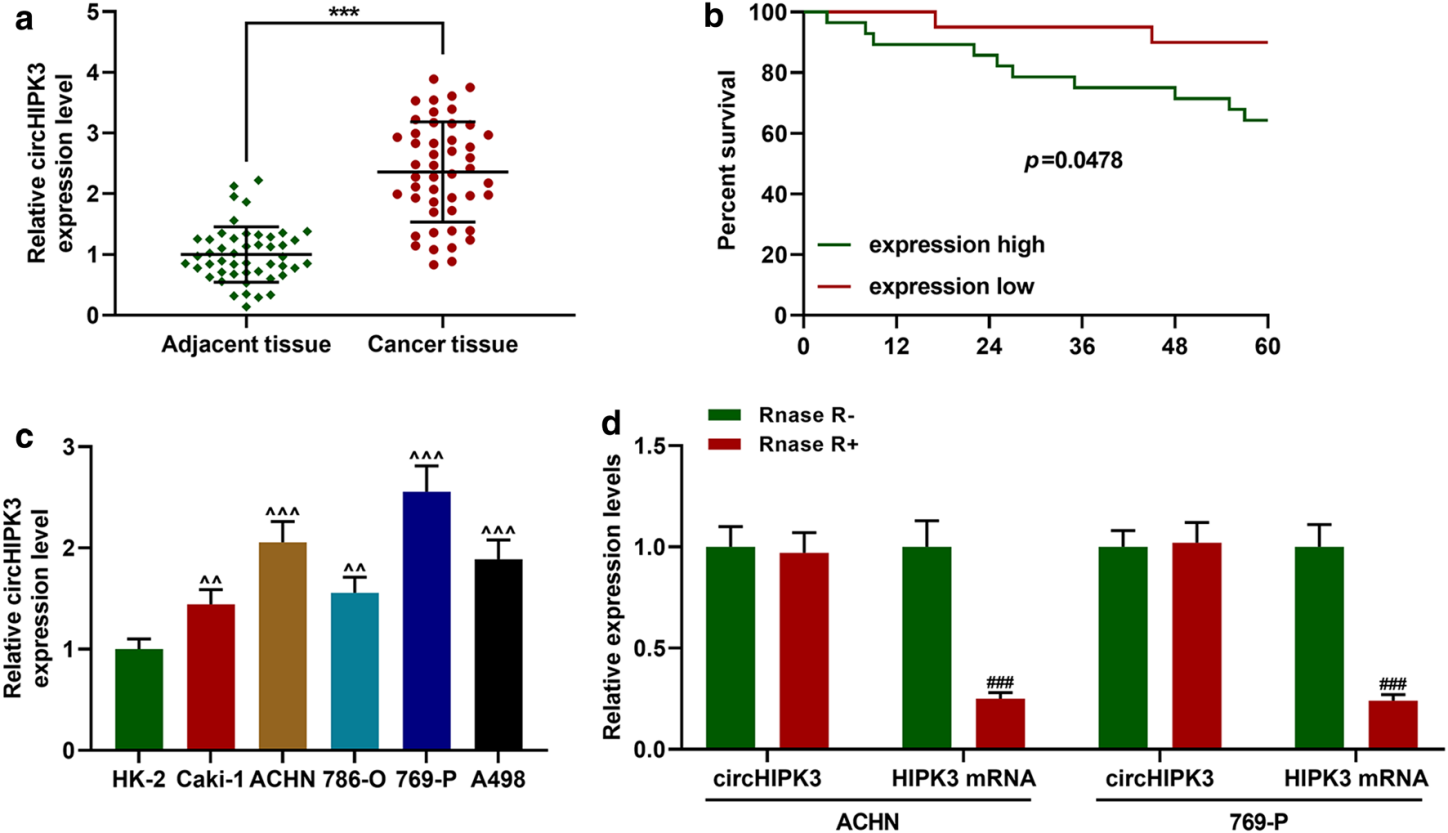
CircHIPK3 was up-regulated in RC tissues and cells, and not sensitive to RNase R. **a** The expression of circHIPK3 in RC tissues and adjacent tissues were measured by qRT-PCR (n = 48). **b** The survival curves were showed. **c** The expression of circHIPK3 in normal renal tubular epithelial cells HK-2 and RC cells (Caki-1, ACHN, 786-O, 769-P, A498) was measured by qRT-PCR. GAPDH was served as an internal reference. **d** MRNA expressions of circHIPK3 and HIPK3 after RNase R treatment were measured by qRT-PCR. ^***^p < 0.001 vs Adjacent tissue; ^^^^*p *< 0.01, ^^^^^*p *< 0.001 vs HK-2; ^###^*p *< 0.001 vs Rnase R-. Rnase R: Ribonuclease R; qRT-PCR: Quantitative Real Time-Polymerase Chain Reaction; GAPDH: Glyceraldehyde-3-phosphate dehydrogenase

The expression of circHIPK3 in RC tissues (adjacent tissues) and RC cells (normal renal tubular epithelial cells) were detected, and the results showed that the expression of circHIPK3 in RC tissues was significantly higher than that in adjacent tissues (Fig. 1A, *p *< 0.001). Figure 1B shows the survival curves of high expression of circHIPK3 (higher than median expression) and low expression of circHIPK3 (lower than median expression), and reveals that patients with low expression of circHIPK3 had higher survival rates than those with low expression of circHIPK3. Moreover, the mRNA levels of circHIPK3 in various cancer cells (Caki-1, ACHN, 786-O, 769-P, A498) were up-regulated (Fig. 1b, *p *< 0.01). Figure 1b demonstrated that circHIPK3 had the highest expression level in ACHN and 769-P RC cells, and thus, the two cells were used for subsequent experiments. RNase R was used to digest and extract RNAs from RC cells because it had no effect on circular RNA despite the fact that it could digest linear RNA. The results showed that there was no significant change in the expression level of circHIPK3 after RNase R treatment, while the mRNA level of HIPK3 was significantly reduced after RNase R digestion (Fig. 1c, *p *< 0.001).

### Knockdown of circHIPK3 inhibited the proliferation, migration, and invasion of RC cells

**Fig. 2:**
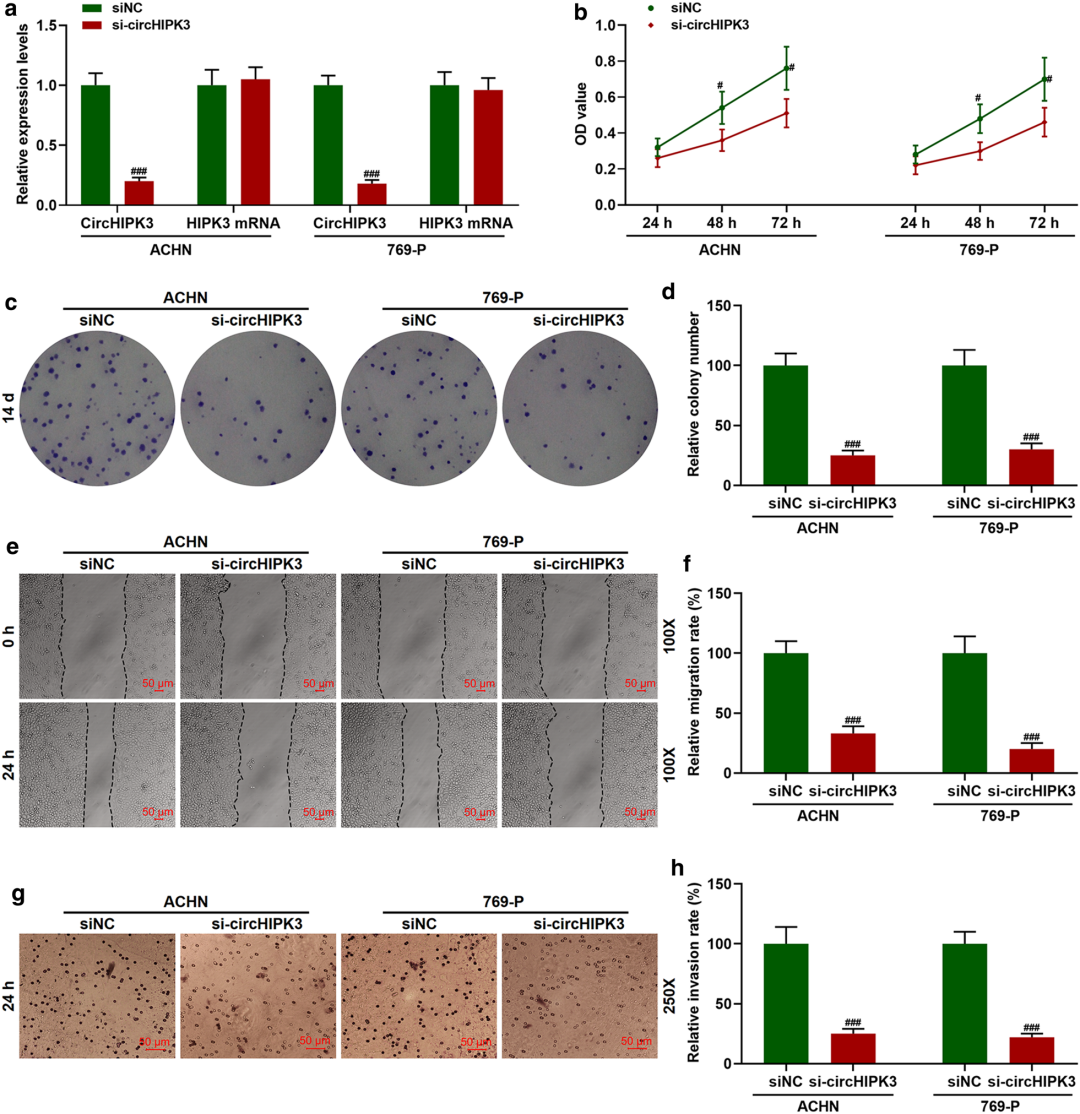
Knocking down circHIPK3 (sicircHIPK3) inhibited the proliferation, migration, and invasion of RC cells. **a** QRT-PCR was used to detect the transfection rate of circHIPK3. GAPDH was served as an internal reference. **b** Cell Counting Kit-8 (CCK-8) assay was used to detect cell viability. **c**, **d** Colony formation assay was used to detect cell proliferation. **e**, **f** Scratch assay was used to detect cell migration (100×). **g**, **h** Transwell assay was used to detect cell invasion (250 ×). ^#^*p *< 0.05, ^###^*p *< 0.001 vs siNC. siNC: negative control for silenced circHIPK3

The expression of circHIPK3 in ACHN and 769-P cells transfected with sicircHIPK3 was significantly inhibited (Fig. 2a, *p *< 0.001). The effect of knocking down circHIPK3 on tumor cell proliferation were detected by CCK-8 and colony formation assay. We found that sicircHIPK3 inhibited the viability (Fig. 2b, *p *< 0.05) and proliferation (Fig. 2c, d, *p *< 0.001) of ACHN and 769-P cells. Moreover, the results of scratch assay indicated that knocking down circHIPK3 significantly inhibited the migration of RC cells (Fig. 2e, f, *p *< 0.001). Consistently, the results of Transwell assay showed that sicircHIPK3 greatly suppressed the invasion of cancer cells (Fig. 2g, f, *p *< 0.001).

### CircHIPK3 expression was negatively correlated with the expression of its target gene miR-485-3p

**Fig. 3:**
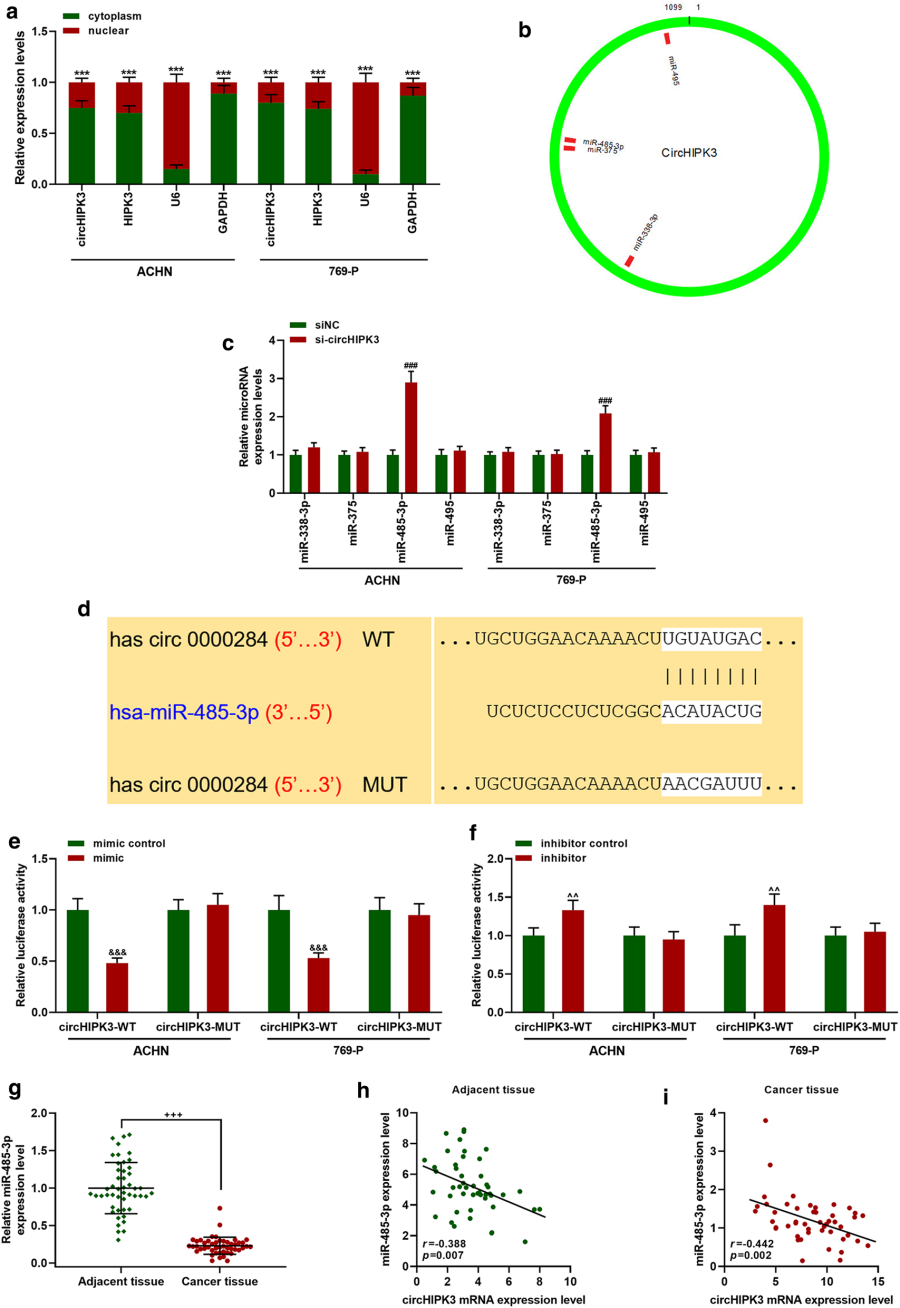
CircHIPK3-targeted miR-485-3p could negatively regulate miR-485-3p. **a** The expressions of circHIPK3 and HIPK3 in the cytoplasm and nucleus were analyzed by qRT-PCR. GAPDH and U6 were used as internal controls. **b** Circular RNA Interactome predicted that circHIPK3 had a targeting relationship with miR-338-3p, miR-375, miR-485-3p, and miR-495. **c** QRT-PCR was used to detect the expressions of miR-338-3p, miR-375, miR-485-3p, miR-495. U6 was used as an internal reference. **d**–**f** Dual-luciferase assay was used to verify the targeting relationship between circHIPK3 and miR-485-3p. **g** The expression of miR-485-3p in RC tissues and adjacent tissues was measured by qRT-PCR (n = 48). **h**, **i** Correlation analysis of circHIPK3 and HIPK3 in RC tissues and adjacent tissues (n = 40). ^***^p < 0.001 vs cytoplasm; ^###^*p *< 0.001 vs siNC; ^&&&^*p *< 0.001 vs MC; ^^^^*p *< 0.01 vs IC; ^+++^*p *< 0.001 vs Adjacent tissues

The expressions of circHIPK3 and HIPK3 in the cytoplasm and nuclei were detected by cytoplasm/nucleus separation experiments. The results revealed that circHIPK3 and HIPK3 mainly existed in the cytoplasm of ACHN and 769-P cells (Fig. 3a, *p *< 0.001). Circular RNA Interactome predicted that circHIPK3 had a targeting relationship with miR-338-3p, miR-375, miR-485-3p, and miR-495 (Fig. 3b). Through detecting the effect of sicircHIPK3 on these miRNAs, we found that sicircHIPK3 significantly up-regulated the expression of miR-485-3p (Fig. 3c, *p *< 0.001), while it had no significant regulatory effect on other miRNAs. Figure 3d showed the conserved binding sites for circHIPK3 and miR-485-3p. To further confirm that miR-485-3p can bind to circHIPK3, we constructed a luciferase reporter vector containing a 3′-untranslation region (3′-UTR). After co-transfection, miR-485-3p mimic reduced the luciferase activity of circHIPK3-WT (Fig. 3e, *p *< 0.01), while miR-485-3p inhibitor increased the luciferase activity (Fig. 3f, *p *< 0.01), indicating that miR-485-3p was a target gene of circHIPK3. We further detected the expression of miR-485-3p in RC tissues, and the results demonstrated that miR-485-3p was greatly low-expressed in RC tissues (Fig. 3g, *p *< 0.001). Correlation analysis results suggested that the expressions of miR-485-3p and circHIPK3 in adjacent tissues (Fig. 3h, *r *= − 0.388, *p *< 0.01) and RC tissues (Fig. 3I, *r *= − 0.442, *p *< 0.01) were negatively correlated.

### Overexpressed circHIPK3 partially reversed the inhibitory effect of miR-485-3p mimic on the proliferation and metastasis of RC cells

**Fig. 4:**
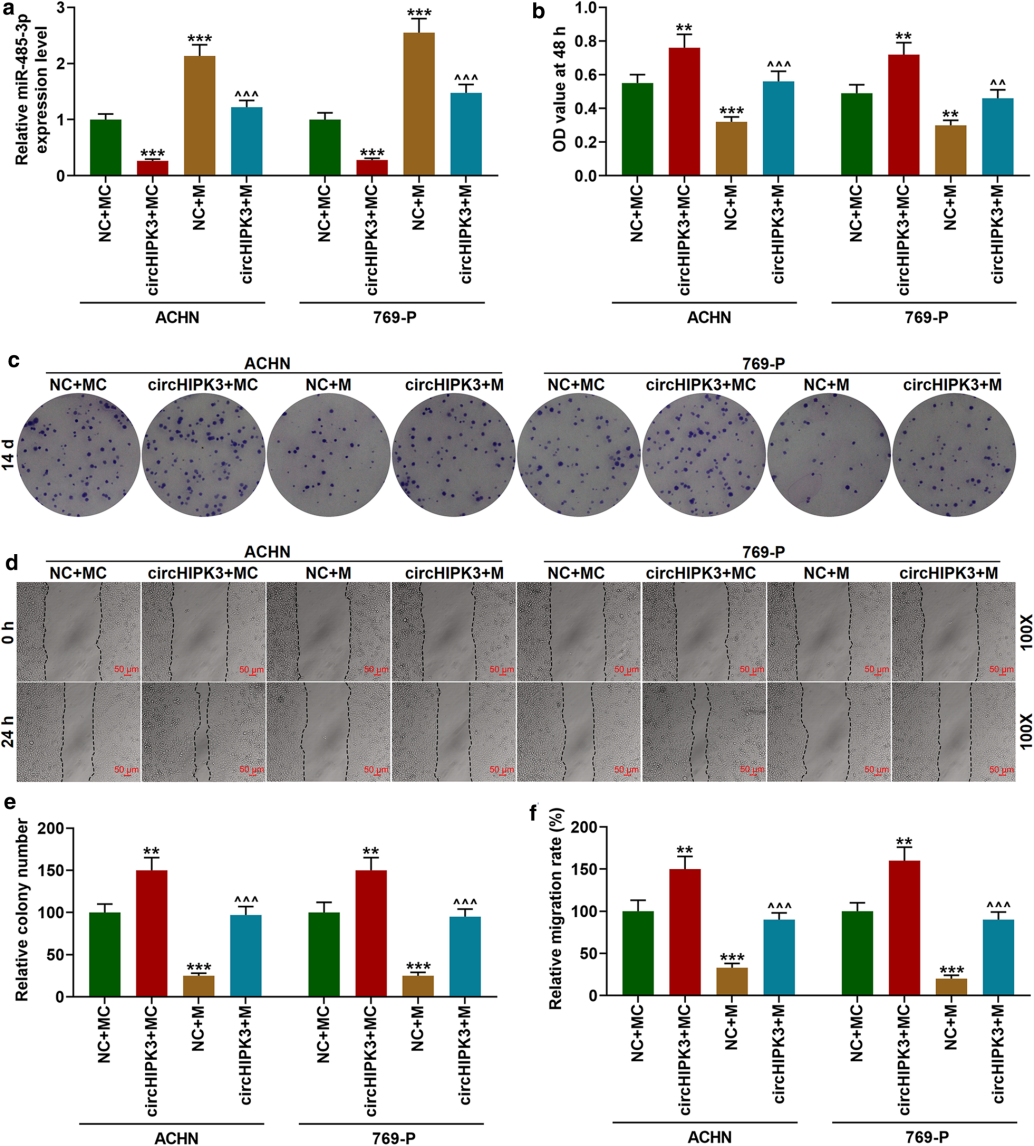
Overexpressed circHIPK3 partially reversed the effect of miR-485-3p mimic on inhibiting the proliferation and metastasis of RC cells. **a** The expression of miR-485-3p was detected by qRT-PCR. U6 was used as an internal reference. **b** CCK-8 assay was used to detect cell viability. **c**, **e** Colony formation assay was used to detect cell proliferation. **d**, **f** The scratch assay was used to detect cell migration (100×). ^**^p < 0.01, ^***^p < 0.001 vs NC + MC; ^^^^*p *< 0.01, ^^^^^*p *< 0.001 vs NC + M. NC: negative control for circular RNA. MC: mimic control; M: miR-485-3p mimic

After co-transfection with miR-485-3p mimic and over-expressed circHIPK3 into RC cells, the expression of miR-485-3p in the cells was inhibited by circHIPK3, while the effect was partially reversed by miR-485-3p (Fig. 4a, *p *< 0.001). In addition, overexpressed miR-485-3p significantly inhibited the viability, proliferation, and migration of ACHN and 769-P cells (Fig. 4b–f, *p *< 0.01), while circHIPK3 partially reversed the effect (Fig. 4b–f, *p *< 0.01). The above experimental results suggested that circHIPK3 can partially reverse the inhibitory effect of overexpressed miR-485-3p on the proliferation and migration of RC cells.

### Overexpressed circHIPK3 partially reversed the effect of miR-485-3p mimic on inhibiting the invasion and promoting the apoptosis of RC cells

**Fig. 5:**
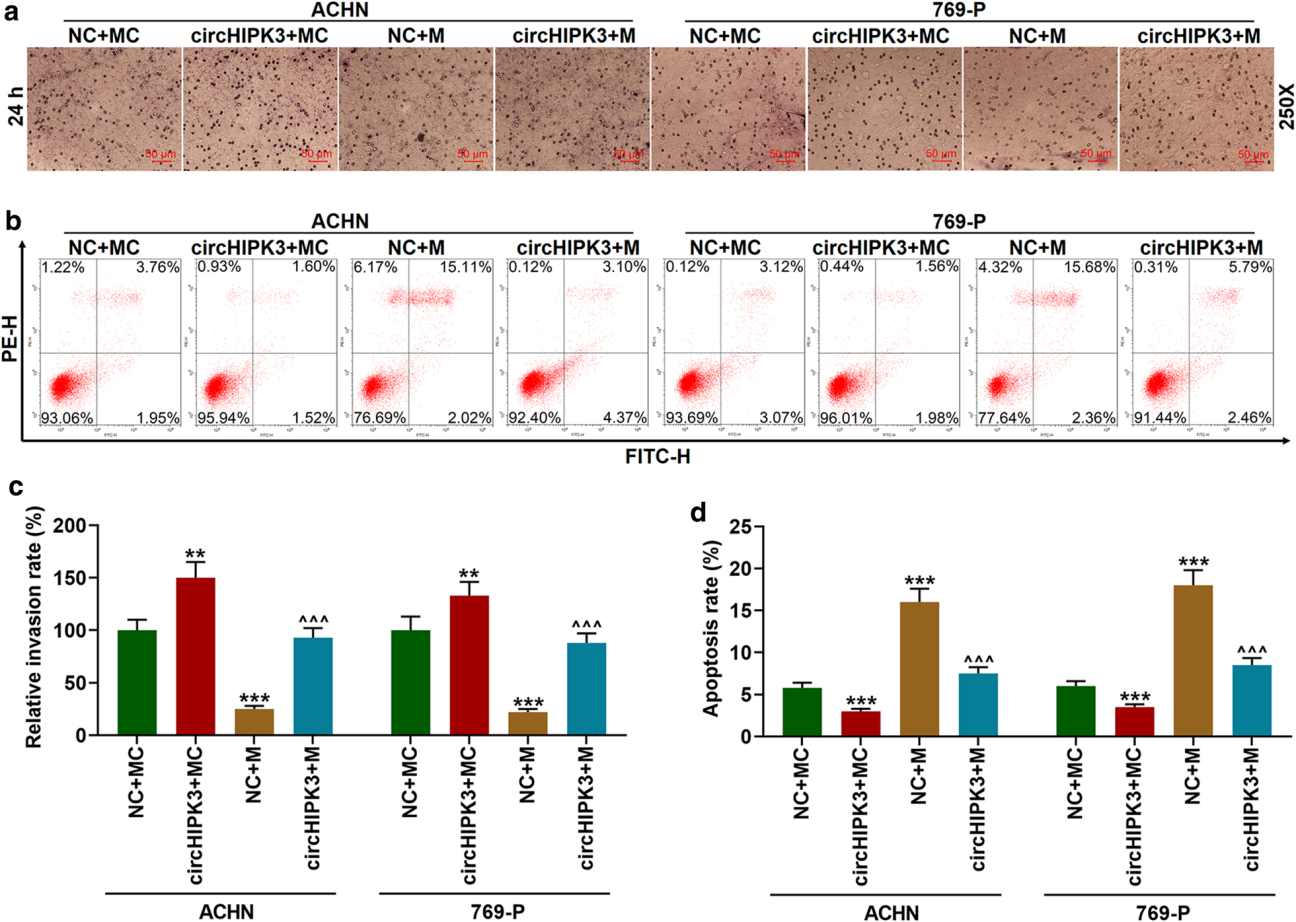
Overexpressed circHIPK3 partially reversed the effect of miR-485-3p mimic on inhibiting the invasion and promoting the apoptosis of RC cells. **a**, **c** Transwell assay was used to detect cell invasion (250×). **b**, **d** Flow cytometry was used to detect apoptosis. ^**^p < 0.01, ^***^p < 0.001 vs NC + MC; ^^^^^*p *< 0.001 vs NC + M. NC: negative control for circular RNA. MC: mimic control; M: miR-485-3p mimic

The effects of miR-485-3p mimic and overexpressed circHIPK3 on the invasion and apoptosis of RC cells were investigated. Transwell assay results showed that miR-485-3p mimic inhibited the invasion of cancer cells, while circHIPK3 partially reversed this effect (Fig. 5a, c, *p *< 0.01). The results of flow cytometry detection showed that the apoptosis of RC cells was increased significantly after miR-485-3p mimic intervention, while the effect was reversed after co-intervention with circHIPK3 (Fig. 5b, d, *p *< 0.001).

### CircHIPK3 promoted apoptosis and inhibited epithelial-mesenchymal transition, and partially reversed the effect of miR-485-3p by regulating associated genes

**Fig. 6:**
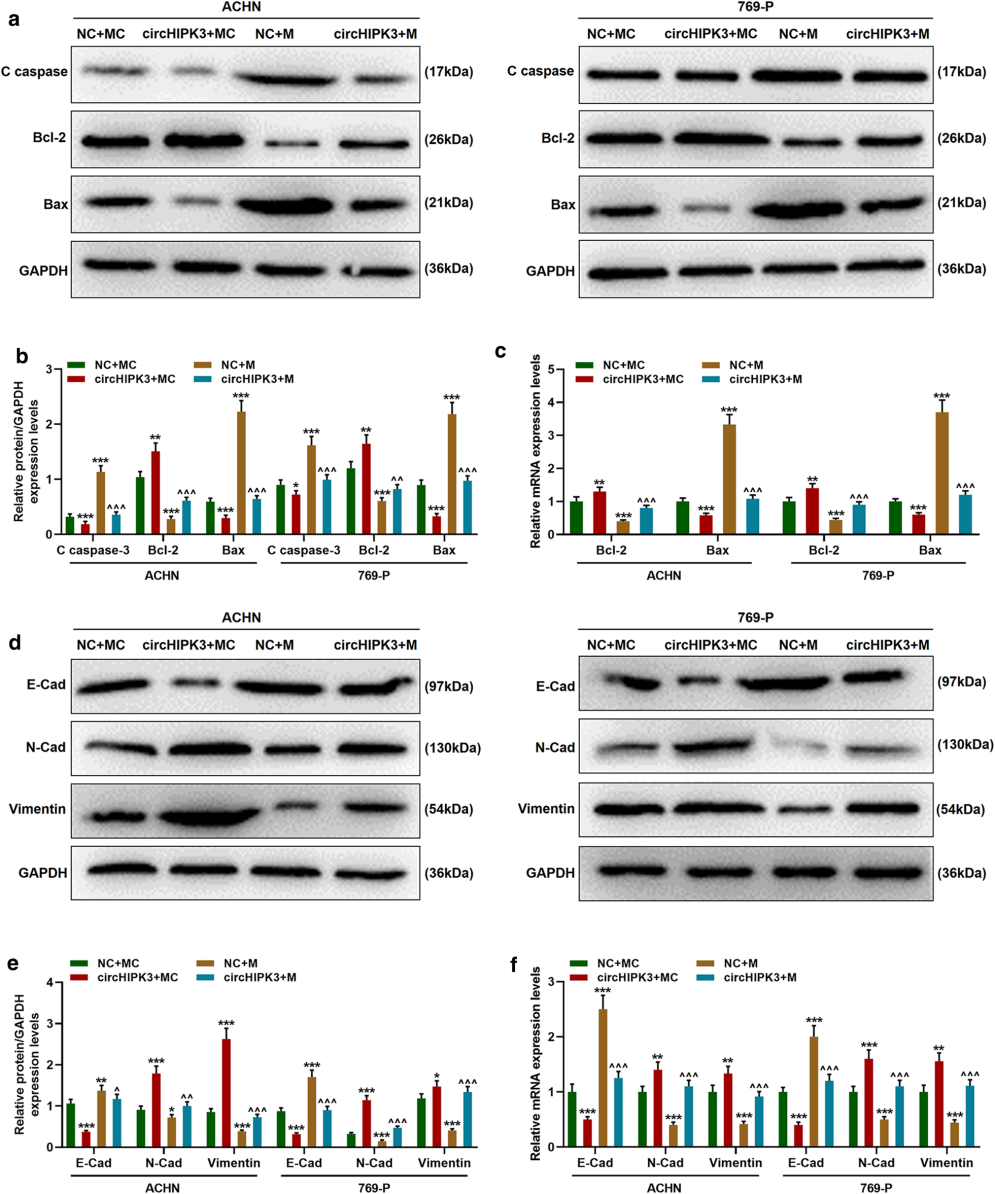
CircHIPK3 partially reversed the effect of miR-485-3p by regulating associated genes to promote apoptosis and inhibit epithelial-mesenchymal transition. **a**, **b** The changes of protein expressions of C caspase-3, Bax and Bcl-2 were detected by Western blot. **c** MRNA expression changes of Bax and Bcl-2 were detected by qRT-PCR. **d**, **e** The expression changes of E-Cad, N-Cad, Vimentin protein were detected by Western blot. **f** MRNA expression changes of E-Cad, N-Cad, and Vimentin were detected by qRT-PCR. ^*^p < 0.05, ^**^p < 0.01, ^***^p < 0.001 vs NC + MC; ^^^^*p *< 0.01, ^^^^^*p *< 0.001 vs NC + M. GAPDH was used as an internal reference. C caspase-3: Clever caspase-3; E-Cad: E-Cadherin; N-Cad: N-Cadherin. NC: negative control for circular RNA. MC: mimic control; M: miR-485-3p mimic

The expressions of apoptosis-associated genes (C caspase-3, Bax, Bcl-2) and epithelial-mesenchymal transition-associated genes (E-Cad, N-Cad, Vimentin) were determined to explore the mechanism of circHIPK3 by which it promoted the metastasis and inhibited the apoptosis of ACHN and 769-P cells. As shown in Fig. 6, miR-485-3p mimic increased the expressions of C caspase-3, Bax, E-Cad, and reduced the expressions of Bcl-2, N-Cad and Vimentin (Fig. 6a–f, *p *< 0.001). However, circHIPK3 not only produced an opposite regulatory effect on these genes, but also partially reversed the regulatory effect of miR-485-3p (Fig. 6a–f, *p *< 0.001).

### MiR-485-3p mimic inhibited tumor growth by regulating apoptosis-associated genes, while circHIPK3 partially reversed the effect of miR-485-3p mimic

**Fig. 7:**
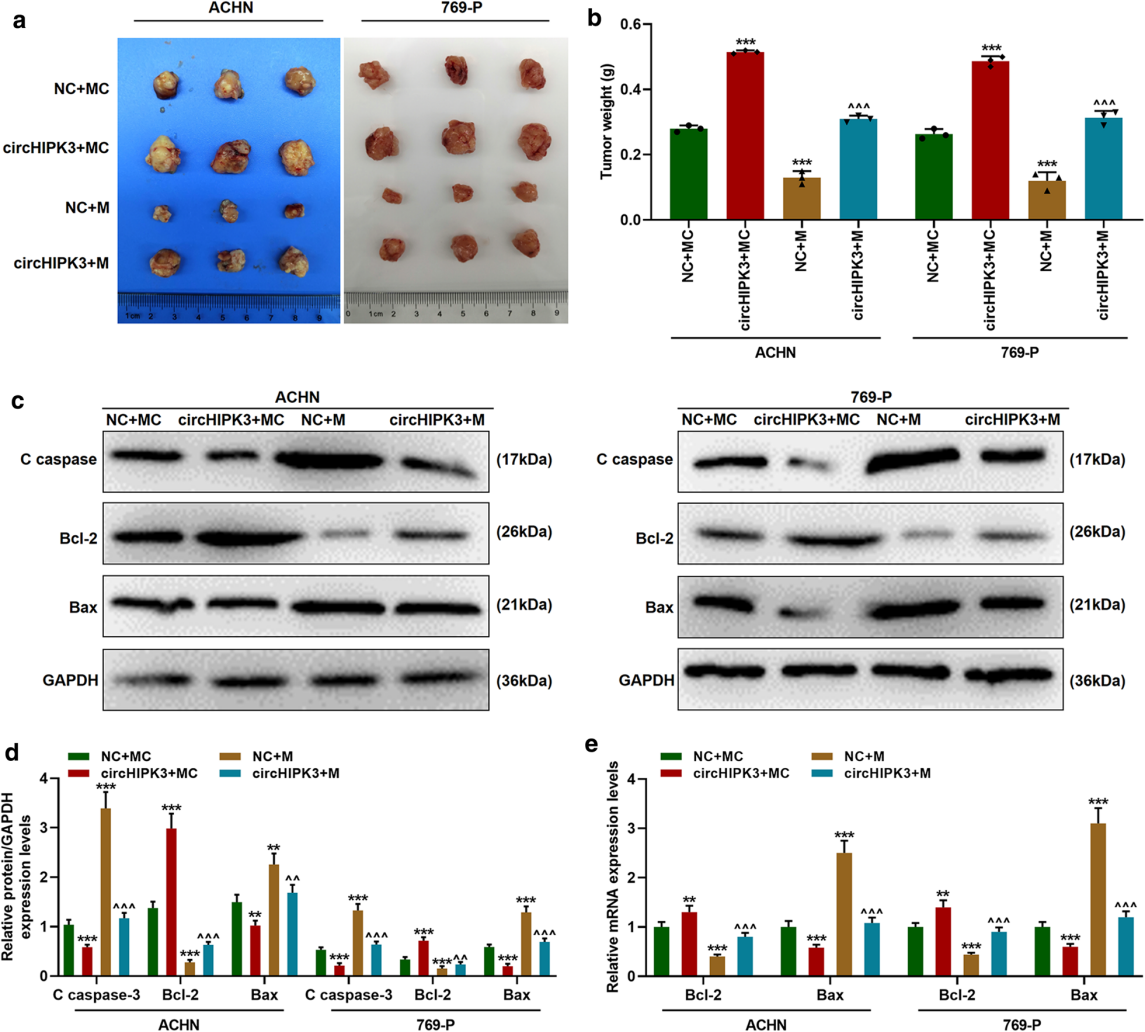
MiR-485-3p mimic inhibited tumor growth by regulating apoptosis-related genes, while circHIPK3 partially reversed the effect. **a** Morphology of transplanted tumor. **b** The weight of the transplanted tumor. **c**–**d** Changes of protein expressions of C caspase-3, Bax, and Bcl-2 were detected by Western blot. GAPDH was used as an internal reference. **e** MRNA expression changes of Bax and Bcl-2 were detected by qRT-PCR. GAPDH was used as an internal reference. ^**^p < 0.01, ^***^p < 0.001 vs NC + MC; ^^^^*p *< 0.01, ^^^^^*p *< 0.001 vs NC + M. NC: negative control for circular RNA. MC: mimic control; M: miR-485-3p mimic

Nude tumor formation experiments were conducted to investigate the effects of miR-485-3p mimic and over-expressed circHIPK3 on tumor growth in vivo. From the transplanted tumor shown in Fig. 7a, we observed that compared with the control group, the tumor diameter of the miR-485-3p mimic group was significantly reduced, and the tumor weight was lighter (Fig. 7a, b, *p *< 0.001). However, the diameters and weights of the transplanted tumors in the CircHIPK3 group were longer and heavier than those in the control group (Fig. 7a, b, *p *< 0.001). Besides, circHIPK3 was found to partially reverse the inhibitory effect of miR-485-3p on tumor growth (Fig. 7a, b, *p *< 0.001). Moreover, we detected the expression changes of apoptosis-associated genes C caspase-3, Bax, and Bcl-2 in transplanted tumors and found that, consistent with the results of cell experiments, miR-485-3p mimic up-regulated the protein levels of C caspase-3, Bax and down-regulated that of Bcl-2 (Fig. 7c, d, *p *< 0.01), and the mRNA levels of Bax and Bcl-2 showed similar changes (Fig. 7e, *p *< 0.01), while circHIPK3 reversed the effect of miR-485-3p (Fig. 7c–e, *p *< 0.01).

### CircHIPK3 partially reversed the inhibitory effect of miR-485-3p mimic on Ki-67 by inhibiting the expression of miR-485-3p

**Fig. 8:**
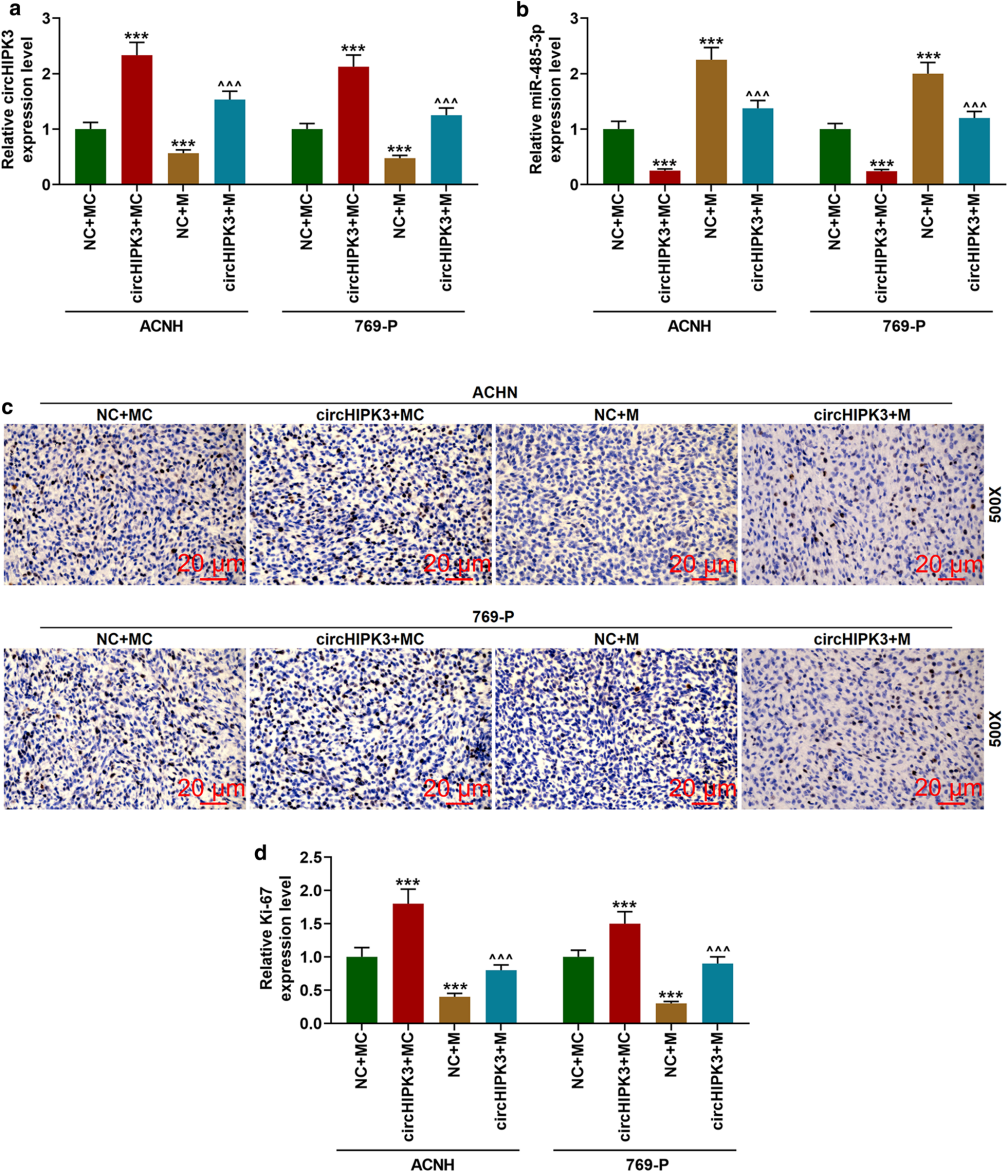
CircHIPK3 partially reversed the inhibitory effect of miR-485-3p on Ki-67 by inhibiting miR-485-3p expression. **a** The expression of circHIPK3 was detected by qRT-PCR. GAPDH was used as an internal reference. **b** The expression of miR-485-3p was detected by qRT-PCR. U6 was used as an internal reference. **c** Immunohistochemistry was used to detect Ki-67 expression in tissues (500×). ^***^p < 0.001 vs NC + MC; ^^^^^*p *< 0.001 vs NC + M. NC: negative control for circular RNA. MC: mimic control; M: miR-485-3p mimic

The expressions of circHIPK3 and miR-485-3p in transplanted tumors were detected, and it was found that overexpressed circHIPK3 up-regulated the mRNA level of circHIPK3 and down-regulated the expressions of miR-485-3p in the transplanted tumor tissues (Fig. 7a, b, *p *< 0.001). Immunohistochemistry was conducted to determine the expression of Ki-67 in the tumor tissues. The Ki-67 positive signal was a brown particle and localized in the nucleus. As shown in Fig. 8c, compared with the control group, the number of brown particles in the CircHIPK3 overexpression group was significantly increased, while it was noticeably reduced in the miR-485-3p mimic group. CircHIPK3 and miR-485-3p mimic affected the positive expression of Ki-67 more significantly in transplanted tumor tissues than in the miR-485-3p mimic group.

## Discussion

Our research showed that circHIPK3, which mainly existed in the cytoplasm, was high-expressed in RC tissues and cells, and that knocking down circHIPK3 could inhibit the proliferation, migration and invasion of RC cells. Previous reports revealed that circHIPK3 is high-expressed in the tissues and cells of colon carcinoma, non-small cell lung carcinoma, and nasopharyngeal carcinoma [25–27]. However, Li et al. [28] found that circHIPK3 is significantly down-regulated in bladder carcinoma cells and is negatively related to the classification, invasion of bladder carcinoma and lymph node metastasis. Thus, these studies indicated that the expression of circHIPK3 differs in various carcinomas. We further studied the mechanism of circHIPK3 on promoting RC, and found that it is closely related to the promotion of carcinoma cell proliferation and EMT, the down-regulation of the expressions of C caspase-3, Bax and E-Cad, and the up-regulation of the expressions of Bcl-2, N-Cad, Vimentin, and Ki-67. C caspase-3, Bax and Bcl-2 are apoptosis-regulating genes [29]. After the apoptotic program is initiated, the anti-apoptotic gene Bcl-2 is inhibited, while Bax gene becomes abnormally active, and the expression of C caspase-3 regulated by Bcl-2 is up-regulated [30]. Zhang et al. [25] found that the expression of Bcl-2 in carcinoma cells is up-regulated by circHIPK3. However, the regulatory effect of circHIPK3 on the above genes differed in various cells. CircHIPK3 was overexpressed in HCM cells with myocardial ischemia/reperfusion injury, and it up-regulated the expression of Bax and down-regulated the expression of Bcl-2 [31]. Both E-Cad and N-Cad are EMT-associated markers, and the conversion from E-Cad to N-Cad is an important mechanism underlying tumor cell metastasis [32]. Vimentin is a type III intermediate fibrin expressed in interstitial cells, and an important cytoskeleton protein [33]. Consistent with our experimental results, Liu et al. [34] reported that knocking down circHIPK3 down-regulated the protein expression of Vimentin and up-regulated the expression of E-Cad.

In this study, miR-485-3p exhibited a high anti-carcinoma activity. It was found that the expression of miR-485-3p was negatively correlated with the expression of circHIPK3, and miR-485-3p could partially reverse the tumor-promoting effect of circHIPK3. Studies showed that miR-485-3p and miR-485-5p, two mature forms of miR-485, reduced the metastasis of breast carcinoma cells by inhibiting the expression of PGC-1α [35], and that miR-485-3p expressed in serum suggested a poor prognosis for glioblastoma patients [36]. Gu et al. [37] also explored the key role of miR-485-3p in the differentiation or proliferation of neural stem cells through regulating TRIP6. MiR-485-3p was reported to also intervene with the angiogenesis of human microvascular endothelial cells and vascular remodeling in chronic obstructive pulmonary disease [38, 39]. From the above studies, it can be found that miR-485-3p possesses an important regulatory effect on carcinoma and other diseases. Our research revealed that miR-485-3p can improve RC. Moreover, the current study for the first time demonstrated that miR-485-3p had significantly low expression in RC tissues and cells, and it could inhibit the proliferation, migration and invasion of ACHN and 769-P carcinoma cells and promote the apoptosis of carcinoma cells.

The relationship between miR-485-3p and circHIPK3 was also investigated in the current study. CircRNAs and miRNAs are both important components of non-coding RNAs. In tumor research, circRNAs serve as the “sponge bodies” of miRNAs, and the mechanism of circRNA on regulating downstream target genes has been widely reported [40, 41]. CircRNAs utilize their own miRNA binding sites as the “sponge bodies” of RNAs to adsorb miRNAs, and regulate the expressions of downstream target genes of miRNAs via competing endogenous RNAs (ceRNAs) [42]. Previous studies revealed that circHIPK3 can function as a sponge body of miRNAs [19, 43, 44], which is consistent with our findings. We further found that the role of miR-485-3p targeted by circHIPK3 in promoting RC is closely related to cell apoptosis and EMT. In addition, miR-485-3p promoted cell apoptosis and EMT by increasing the expressions of C caspase-3, Bax and E-Cad, and decreasing the expressions of Bcl-2, N-Cad, Vimentin, and Ki-67, thus reversing the effect of circHIPK3.

Nevertheless, there were some limitations to this study. For example, ACHN and 769-P RC cells used in this study were not clear RC cells.

## Conclusion

In conclusion, circHIPK3 was high-expressed in RC tissues and cells. CircHIPK3 promoted tumor growth, proliferation and metastasis and inhibited apoptosis of RC cells by inhibiting the expressions of C caspase-3, Bax and E-Cad and promoting the expressions of Bcl-2, N-Cad, Vimentin and Ki-67. MiR-485-3p produced a significant anti-carcinoma effect, while the effect was partially reversed by CircHIPK3. The current study filled up the gap of circHIPK3 intervention in RC and provided a basis for molecular-targeted therapy for RC.

## Data Availability

The analyzed data sets generated during the study are available from the corresponding author on reasonable request.

## Abbreviations

RC: Renal carcinoma
qRT-PCR: Quantitative real-time polymerase chain reaction
RNase R: Ribonuclease R
CCK-8: Cell counting kit-8
EMT: Epithelial-mesenchymal transition
HIPK3: Homeodomain-interacting protein kinase 3
RNase R: Ribonuclease R
GAPDH: Glyceraldehyde-3-phosphate dehydrogenase
